# School Safety Concerns and Solutions: A Qualitative Analysis of U.S. School Psychologists’ Perspectives

**DOI:** 10.3390/bs15020228

**Published:** 2025-02-18

**Authors:** Luz E. Robinson, Kate R. Watson, Natalie Fensterstock, Sawyer Hogenkamp, Yinuo Xu, Hannah Garner, Vanessa R. Warri, Casie H. Morgan, Anthony A. Garcia, Chaoyue Wu, Danielle Dunn, Dorothy L. Espelage, Ron A. Astor, Susan D. McMahon, Linda A. Reddy, Eric M. Anderman, Frank C. Worrell, Andrew Martinez

**Affiliations:** 1 Department of Psychiatry and Behavioral Sciences, Medical University of South Carolina, Charleston, SC 29425, USA; 2 School of Education, University of North Carolina at Chapel Hill, Chapel Hill, NC 27599, USA; 3 Department of Social Welfare, University of California-Los Angeles, Los Angeles, CA 90095, USA; 4 Graduate School of Education, University of Pennsylvania, Philadelphia, PA 19104, USA; 5 School of Education and Information Studies, University of California, Los Angeles, CA 90095, USA; 6 Department of Psychology, DePaul University, Chicago, IL 60614, USA; 7 Graduate School of Applied and Professional Psychology, Rutgers University, New Brunswick, NJ 08901, USA; 8 Department of Educational Studies, The Ohio State University, Columbus, OH 43210, USA; 9 Berkeley School of Education, University of California-Berkeley, Berkeley, CA 94720, USA; 10 New York Center for Justice Innovation, New York, NY 10018, USA

**Keywords:** school violence, school psychologists, qualitative, school safety

## Abstract

In the present study, we analyzed qualitative survey data from 538 school psychologists across schools in the United States regarding their perceptions of school safety issues and potential strategies to address school safety. There are only a few studies exploring the experiences and perspectives of school psychologists that have been based on large-scale qualitative data. Using inductive coding, three themes for safety concerns emerged: (a) aggressive behaviors from students, (b) mental and behavioral needs, and (c) limited staffing. Three themes also emerged for potential solutions: (a) professional development/training, (b) school–family–community relationships, and (c) threat assessments. These findings from school psychologists have implications for addressing structural issues to prevent school violence in research and practice. Policy recommendations to inform resource allocation and improve school safety are discussed.

## 1. Introduction

School safety remains a critical concern in the United States, with widespread attention focused on the alarming rates of violence in educational settings (Katic et al., 2020; Polanin et al., 2021). From physical altercations between students to threats and aggression directed at staff, the school environment is reflecting broader societal issues, including violence, inequities, and strained resources in a system that is overwhelmed (Astor & Benbenishty, 2019; Astor et al., 2021, 2023). In response to safety concerns over the past decade, a variety of school safety interventions for students and school staff have been proposed and implemented at local, state, and federal levels. Specifically, most school-based interventions focus on crisis response strategies, such as lockdown drills (Schildkraut & Nickerson, 2020; Schildkraut et al., 2023; Schonfeld et al., 2020), threat assessments (D. Cornell et al., 2018), and the increased presence of School Resource Officers (SROs; D. G. Cornell et al., 2020).

These approaches, however, are often reactive and disproportionately affect marginalized students, who may already be over-policed within their communities (Flannery et al., 2021; Zimmerman & Astor, 2021). Furthermore, many interventions are informed by policymakers and law enforcement agencies, rather than by the educators and mental health professionals who are directly involved in students’ day-to-day experiences (National Institute of Justice, n.d.; Peterson, 2021; U.S. Department of Education, 2014). Outcome data on these safety measures suggest that they have limited effectiveness, particularly when schools fail to address root causes of violence, such as inequitable access to mental health services and the broader social determinants of safety (Espelage et al., 2023). The National Institute of Justice has highlighted a comprehensive school safety approach and the importance of equity-centered solutions that prioritize the well-being of all students, including those from marginalized communities, yet such approaches remain underutilized (Espelage et al., 2023).

According to data from the 2023 report on Indicators of School Crime and Safety (Irwin et al., 2024), several crime and safety issues have become less prevalent in K–12 school settings compared to a decade earlier (e.g., Benbenishty et al., 2023). Specifically, incidents of nonfatal student and teacher victimization, including bullying and theft, decreased significantly from 2012 to 2022 (Irwin et al., 2024). However, these findings must be interpreted with caution, as is suggested in the report, given the context in which the latest data were collected. During much of the COVID-19 pandemic (2020–2022), many students were learning remotely or attending school part-time through hybrid models, which likely contributed to the decline in reports of school violence. With fewer students physically present in schools, opportunities for violence decreased, and the data from this period may not fully reflect the current reality of school safety concerns post-pandemic.

With this in mind, some concerning trends have still emerged. Public schools reported higher incidence rates of firearm possessions compared with the early 2010s (Irwin et al., 2024). Despite lower rates of students bringing weapons onto school property being observed in recent years, during the 2021–2022 school year, 5000 U.S. public school students from kindergarten to 12th grade were reported to have possessed firearms (Irwin et al., 2024). This indicates an overall rate of 10 firearm possessions per 100,000 students, which is higher than in any other school year over the previous decade (ranging from 2 to 7 possessions per 100,000 students). Further, there were four active shooter incidents documented at elementary and secondary schools in 2022 that resulted in 52 casualties, the second highest number in any year since 2000, following 2018, with 81 casualties (Irwin et al., 2024).

Across the U.S., a majority of schools are indicating increases in violent/disruptive behaviors directed against students and staff since the COVID-19 pandemic (Da et al., 2023; Effective School Solutions, 2022; Vaillancourt et al., 2021). In 2021–2022, U.S. public schools indicated that several student behaviors increased since returning to in-person instruction from COVID-19: physical attacks or fights between students, bullying, vandalism, classroom disruptions from student misconduct, and threats, violence, or disrespect toward school staff members (National Center for Education Statistics, 2024). The prevalence of these incidents has underscored the need for effective interventions that address both immediate safety concerns and the underlying systemic factors that contribute to them.

School psychologists are one group of professionals responsible for fostering and promoting a positive and safe learning environment. According to the website of the National Association of School Psychologists [NASP] (n.d.), school psychologists “apply expertise in mental health, learning, and behavior, to help children and youth succeed academically, socially, behaviorally, and emotionally” (para. 1). By collaborating with families and school personnel (i.e., teachers, staff, and administrators), school psychologists contribute to school safety by supporting and implementing evidence-based practices, including bullying prevention and social–emotional learning programs and strategies, to improve student learning and mental health outcomes (National Association of School Psychologists [NASP], n.d.). Given school psychologists’ critical role in assessing and ensuring psychological and physical safety (Bradshaw et al., 2021; Morrison et al., 1994), their perspectives on school safety are essential to enhancing our current understanding of the challenges in schools to effectively address them. Further, given the complex factors that influence school safety, there is a critical need for qualitative data from U.S. school psychologists that captures problems and solutions to advise school policy. However, although there have been several studies documenting the perspectives of school psychologists on various issues, such as COVID-19 restrictions (May et al., 2023) and social justice (Shriberg et al., 2011), there is a need for qualitative studies to more deeply understand their school safety concerns and recommendations.

Prior studies report that school psychologists face verbal abuse, threats, and even physical harm from both students and parents as they attempt to intervene in escalating behavioral issues (Ferrara et al., 2019). Emerging data suggest that the COVID-19 pandemic has only exacerbated existing challenges to school safety (e.g., Da et al., 2023; Effective School Solutions, 2022; Vaillancourt et al., 2021). As schools transitioned to virtual learning, investigations found that many students faced unprecedented adverse childhood experiences, isolation, disrupted routines, and increased exposure to household stressors (Levitt et al., 2022). These experiences have been linked to a rise in mental health crises and aggressive behaviors (Centers for Disease Control and Prevention, 2021). Upon returning to in-person learning, school staff have observed a marked deterioration in both student behavior and parent–school relationships. With many students lacking self-regulation skills and parents frustrated by the challenges of the pandemic, school staff have found themselves navigating more volatile and unpredictable situations (e.g., Da et al., 2023; Effective School Solutions, 2022; Vaillancourt et al., 2021).

The current qualitative investigation aims to address the possibility that school psychologists experience an increased likelihood to be negatively impacted by the relationship between school climate and safety, student–family dynamics, and broader community tensions. We reason that the stressors that emerged post-pandemic elevated negative mental health outcomes and may increase this likelihood. As observed in the pandemic, aggression in adults increased during COVID-19 lockdowns (Killgore et al., 2021), and the internal tensions and external stressors during this period may have posed risks of an increase in harsh parenting in parent–adolescent relationships (Harms & Record, 2023). As research has indicated that bullying in school contexts is positively associated with sibling aggression and parenting characteristics such as harsh discipline (Tippett & Wolke, 2015), we argue that the associations among school safety and climate, student family dynamics, and community tensions warrant investigation. Given their role within school environments, school psychologists can offer unique and valuable perspectives on these issues (National Association of School Psychologists [NASP], n.d.).

### 1.1. Theoretical Framework

Astor et al. (2023) developed a socioecological model specifically designed to address school safety and violence prevention. This model integrates U.S. culture and power structures, including policy and practice, more directly into the ecological layers, emphasizing how government decisions, resource allocation, and school policies shape the safety environment at both the systemic and school levels. This approach highlights the need for structural interventions to prevent violence, acknowledging that safety concerns are deeply rooted in broader systemic inequities, including resource shortages and inadequate staff training. For this reason, the model situates school policy as a key lever for change, demonstrating how top-down interventions can have ripple effects across all levels of the school environment, from staff to students and families. This model is particularly useful for contextualizing our findings on school psychologist perspectives of school safety concerns and potential policy and practice solutions.

To further inform the methodology and contextualize the findings, we incorporated Espelage’s (2014) person-centered ecological model, which has been used to understand the interconnected factors influencing peer aggression and bullying behaviors from the perspective of the victim or perpetrator. A person-centered approach adapts the traditional socioecological approach by positioning school psychologists at the center of the analysis, emphasizing their direct interactions with students, families, and school staff, while they also navigate broader systemic and cultural forces. In our study, school psychologists act as central agents in identifying and addressing safety concerns, situated within a web of interconnected systems. By framing school psychologists as key actors, we can better understand how their perspectives provide insight into the multi-layered nature of school violence, from interpersonal issues to systemic failures. This framing illustrates the interconnectedness of the socioecological (Astor & Benbenishty, 2019) and person-centered ecological models (Espelage, 2014), with school psychologists at the core, surrounded by various ecological systems that shape safety dynamics—highlighting the reciprocal influences between individual behaviors, school climate, family engagement, and larger policy environments.

### 1.2. Present Study

To understand school safety issues and potential solutions, we analyze qualitative survey data collected during 2021–2022 from school psychologists across pre-K–12 schools (i.e., students aged 4–18) in the United States. This study focuses on two research questions. First, what are the biggest safety issues facing educators and staff in your school? Second, what policies, procedures, or interventions are needed to better prevent or address violence in your school? Using a socioecological framework, the school psychologists’ role and their perspectives are centered at the individual level to capture the interconnected nature of challenges and solutions to safety concerns in schools across the U.S.

## 2. Method

### 2.1. Data Collection

The data in this study were collected as part of a project of the American Psychological Association Task Force on Violence against Educators and School Personnel (McMahon et al., 2024). The Task Force collaborated with professional associations affiliated with school personnel, including the NASP. The NASP distributed the survey to school psychologists through emails to a subset of their members. In addition, a subset of school staff emails were obtained from MCH Strategic Data, which were stratified by role (i.e., teacher, administrator, staff member), region (i.e., West, Midwest, South, Northeast), urbanicity (i.e., rural, town, suburban, urban), and school level (i.e., primary/elementary, middle, high school, combined). Data were collected from March 2022 to June 2022, after most COVID-19 restrictions in schools ended.

The data included a series of open- and close-ended questions. This study focuses on two open-ended qualitative questions from the survey: (a) what are the biggest safety issues facing educators and staff in your school, and (b) what policies, procedures, resources, or interventions are needed to better prevent or address violence in your school?

### 2.2. Participants

The participants were 538 school psychologists who answered at least one of the two qualitative questions listed in the previous paragraph. There were 461 school psychologists who responded to question 1; 470 responded to question 2, and 461 answered both questions. Their demographic characteristics are provided in Table 1.

### 2.3. Analytic Approach

Data were analyzed by psychology and social work graduate and undergraduate students with training in qualitative thematic analysis (Braun & Clarke, 2006). The analysis started with inductive coding of a random 10% of the sample. These preliminary findings were used to create a coding frame and manual, including broad categories, codes, and subcodes. This codebook was tested on another randomized 10% of the sample and subsequently refined and finalized. Coders were trained on the finalized codebook using a deductive approach (Braun & Clarke, 2006), which was used for the remainder of the dataset. The entire dataset was coded by six researchers. Three pairs of researchers (*n* = 6) individually coded 33% of the data, and then compared results with their partner.

Inter-coder reliability (ICR) was >80% among the three pairs and determined manually using percent agreement (O’Connor & Joffe, 2020). Differences were resolved using a consensus approach. Once consensus was reached, all quotes were moved to a final coding document. Each member then iteratively reviewed the coded data, identifying patterns of response, and noted key takeaways and reflections. Overall, the analysis generated three major themes for each of the two research questions, with five and three subthemes, respectively.

## 3. Results

### 3.1. Question 1: What Are the Biggest Safety Issues Facing Educators and Staff in Your School?

School psychologists’ school safety responses yielded three major themes: aggressive behaviors from students, mental and behavioral health needs, and staffing concerns. Five additional subthemes emerged based on how commonly they were referred to. Subthemes are nested in the results across the three major themes and include parent/family aggression, community factors, defiance/disruption, staff burnout/role overload and confusion, and being generally safe. The safety concerns expressed by school psychologists appear to be interconnected and reflective of various layers within the social ecology of their schools, encompassing an overwhelmed system responsible for managing student behavioral health, family dynamics, and broader community factors without the proper staffing support in place.

#### 3.1.1. Aggressive Behaviors from Students

The first theme, aggressive behaviors from students, was described by respondents as a significant safety issue. This theme highlights how behaviors, particularly following the COVID-19 pandemic, have deteriorated school safety. One school psychologist responded,

*Students with mental health and special needs leaving the school building, hiding, running away. Also have significant behavior outbursts including aggression and violence toward staff and other students. Keeping everyone safe while meeting the significant mental health needs has been a challenge especially with staff shortages and quarantine requirements due to COVID.*

[P69, Elementary, 26 years’ experience]

At the same time, the parent/family aggression subtheme captures the rising tension between school staff and families, with some parents becoming verbally abusive, adding further complexity to how families impact student behavior. As one school psychologist pointed out, in their school, parent and student aggression are safety concerns:

*Verbally and physically aggressive parents, and parents with a propensity toward violence. The behavior of the students has deteriorated markedly since Covid, but the behavior of many of the parents has just gotten out of hand.*

[P242, Elementary, 19 years’ experience]

In addition, psychologists highlighted how the subtheme community factors contribute to these aggression-related challenges, with the broader neighborhood’s influence on student behavior. One school psychologist noted the impact of neighborhood violence on school safety:

*The neighborhood is the biggest safety issue. We have frequent gang related activities. Incidents have escalated to the point that our school recently had a forced early dismissal and remote instruction for a week due to incidents within the area of the school building.*

[P234, Elementary, 23 years’ experience]

These violent interactions among students, families, communities, and school staff directly affect the safety and well-being of school psychologists and their school communities.

#### 3.1.2. Mental and Behavioral Health Needs

The second theme psychologists described was mental and behavioral health needs. School psychologists reported instances where the lack of access to school mental health professionals compounded unaddressed mental health issues among many students. As one respondent explained,

*Students with diagnosed or undiagnosed/misdiagnosed [Mental Health] MH disorders, demonstrate extreme behavior, violence, physical aggression and staff who aren’t trained to support or deal with it. [There are] too few staff who are trained; we get burnt out and the student is not getting what they need, not learning, not safe, and have used all our resources available to us with little to no improvement. It’s not sustainable.*

[P385, Elementary, 15 years’ experience]

Another school psychologist highlighted their explicit concerns for the prevalence of intrapersonal violence (e.g., self-harm and suicide), which has the potential to spread through peer networks:

*The biggest safety issue we face is students harming themselves. We have had a completed suicide in our school within the past year. There have been many attempts (some that we know of—some that we don’t). Students are harming themselves.*

[P355, Middle School, 21 years’ experience]

Public schools in the U.S. are expected to provide free appropriate public education to students who meet disability criteria through the Individuals with Disabilities Education Act. Another school psychologist described how their schools are not equipped to support students with an Emotional Disturbance disability classification:

*The biggest safety issues in my school(s) are often created by students identified with behavioral/emotional problems. Due to a shortage of trained special education teachers, we have long term substitutes trying to manage ED classrooms and this is not working out well. Violence among this population of students has dramatically increased this year.*

[P533, Elementary, 35 years’ experience]

The *defiance/disruption* subtheme highlights how aggressive behaviors among students with emotional and behavioral disabilities, regardless of their age or grade, are a major school safety concern. One school psychologist detailed their concerns across their three school placements:

*High School: Students with emotional and behavioral disorders who become physically aggressive toward staff when given a non-preferred task or a difficult task without the supports necessary for completing it.*

*Middle School: Students with emotional and behavioral disorders as well as students with a history of antisocial behavior who become defiant, disruptive, and verbally and physically aggressive toward peers and staff.*

*Elementary School: Students who tantrum and become physically aggressive toward peers and staff in response to being given a demand involving a non-preferred task or activity.*

[P92, All Grades, 18 years’ experience]

Students with mental and behavioral health needs navigating a school environment where staff are unable to provide support can create a ripple effect influencing school safety, which in turn increases the burden on school-based services and staff.

#### 3.1.3. Staffing Concerns

The final theme, staffing concerns, reflects systemic issues where institutional policies and resource allocation shape the school environment and safety. Respondents reported concerns about untrained and understaffed school environments, noting that a lack of qualified personnel makes it difficult to address both safety concerns and the growing mental health needs of students. As one respondent emphasized,

*With the increase of mental health needs, the staffing ratios are no longer sufficient and cause staff to be spread too thin to cover the needs, or staff spend all day addressing mental health needs and aren’t able to complete other legally required job duties which in turn causes staff burn out and more shortages. Lack of paraprofessionals to meet the needs of students in the self-contained programs.*

[P481, Elementary, 5 years’ experience]

Another school psychologist shared concerns about inadequate preparation for a crisis:

*We have a crisis plan, but the person in charge is only in our school maybe two days/week. If something were to happen when she is not there, I don’t know that others would know what to do.*

[P38, All Grades, 5 years’ experience]

Along similar lines, one psychologist expressed concern with preparation for school shootings, highlighting increases in youth access to guns and decreases in mental health support:

*My biggest fear is a school shooting incident, although I have no reason to expect it will happen here, it seems to be a rising trend with no legislative changes and a senate paid off by the gun lobby. I also feel we are not adequately equipped to deal with such a crisis both in training and in the building’s limitations. At the same time, I feel active shooter drills are traumatizing, so I’m not sure what the answer is aside from stricter gun control and funding mental health. Aside from guns, some students with behavioral issues put staff at physical risk to a moderate degree.*

[P376, All Grades, 7 years’ experience]

Additionally, the burnout and role overload subtheme described school contexts where insufficient staffing ratios have led to an inability of staff to provide effective intervention and prevention strategies because they are performing tasks beyond their job descriptions. As one psychologist pointed out,

*Due to a lack of resources, student services staff (counselors, social workers, psychologists) are spread too thin. We are unable to provide comprehensive, preventative services. Students have not had access to adequate, high-quality core academic instruction and universal positive behavior support due to learning disruptions. School closures, high rates of staff absences, vacancies due to resignation, and inconsistent bus transportation cause remaining staff to pick up duties that are beyond their primary job responsibilities. For instance, staying hours after the instructional day because students don’t have school transportation due to a missing bus driver.”*

[P498, Middle School, 15 years’ experience]

This shortage leaves staff feeling unsupported, contributing to burnout and hindering the school’s ability to maintain a safe and effective learning environment. Despite these concerns, a portion of respondents indicated that their schools felt generally safe, a subtheme suggesting some variability in perceptions of school safety:

*I don’t feel at the elementary level that we have significant safety concerns in our school.*

[P181, Elementary, 10 years’ experience]

These themes and subthemes reflect the interconnected socioecological nature of safety concerns in schools through the lens of school psychologists. Aggressive behaviors, mental health challenges, and staffing shortages operate at multiple levels within the school’s social ecology, from the individual student to family/community influences to systemic staffing policies. School psychologists are on the front lines, navigating these challenges within a dynamic environment that requires coordinated responses across all levels of the school’s social structure.

### 3.2. Question 2: What Policies, Procedures, Resources, or Interventions Are Needed to Better Prevent or Address Violence in Your School?

School psychologists often responded by listing various interrelated intervention and prevention strategies or describing their current school situations (i.e., policies, procedures, resources). Their responses detailed areas for improvement to enhance school safety and yielded three major themes, including professional development/training, school–family–community relationships, and threat assessment. Three additional subthemes emerged and are nested across the results. The three major subthemes included discipline, school-based mental health, and being generally safe.

#### 3.2.1. Professional Development (PD)/Training

The first theme, professional development (PD)/training, was most prevalent and emphasized school psychologists’ perceptions around a growing need for evidence-based training among all school staff to effectively address and prevent school violence. Specific training needs were identified, including cultural diversity, restorative practices, social–emotional learning (SEL), and trauma-responsive training. One school psychologist expressed it this way:

*Diversity training and awareness for staff, students, and parents/community members. Since we are a rural and primarily white school district, the students (and often community members and sometimes staff) are not very tolerable to individual differences in culture, race, general identity, religion, etc.*

[P468, High School, 19 years’ experience]

Another school psychologist expanded on this need by explaining how changes in school demographics require diversity training to prevent school violence:

*We don’t have a lot of violence. Right now our need is more teaching for the community to understand and accept cultural/racial differences. It’s not a very diverse community, but we’ve recently had increased enrollment from minority groups that require student teaching on differences and acceptance.*

[P481, Elementary, 5 years’ experience]

The subtheme discipline emerged often, with a call for training on de-escalation, conflict resolution, and restorative practices. One respondent shared the need for training that focuses on restoring relationships and repairing harm as consequences to school violence:

*This school has become too quick to put hands on students. We need training and support for de-escalation and we need training and support in restorative practices to repair the harm.*

[P6, Elementary, Missing years’ experience]

Another respondent pointed to the need for cultural responsiveness and cultural humility in educators’ training to reduce inequitable discipline practices. This school psychologist shared,

*Educators need training on their implicit cultural bias toward students (and parents) from different racial, cultural, and economic backgrounds. The White female teachers seem to single out the Black boys as needing discipline and special education…At this particular elementary school, students are “bussed in” from the inner city in order to create more racial diversity and that leads to disproportionality in terms of discipline referrals and special education referrals.*

[P402, Elementary, 18 years’ experience]

There is also a need for schools to dismantle power imbalances that perpetuate the “school to prison pipeline”. One psychologist expands on this by detailing that the student perspective in discipline is needed for accountability, and can be leveraged to develop consequences that are reasonable and effective at addressing school violence:

*Increased value of student voice. Increased understanding of social justice. Dismantling of token economies. Restorative justice. Reasonable rules and meaningful consequences rather than arbitrary punishment.*

[P44, All Grades, 30 years’ experience]

Many psychologists listed the following prevention and intervention practices for school climate at the universal (Tier 1) level, including positive behavioral interventions and supports (PBIS):

*trauma-informed practice, reducing exclusionary discipline and replacing it with more restorative practices, increasing understanding of mental wellness strategies to use in the classroom, increasing school climate for students, increasing effective Tier 1 PBIS practices, not sending a police officer every time a student is out of location in the building.*

[P300, Middle School, 16 years’ experience]

There was also support for crisis prevention and for all staff to use consistent social–emotional learning (SEL) and universal screening to detect and address school violence concerns:

*All staff should be trained in Safety Care practices not just the school psych and few other staff. Additionally, more SEL woven into the day along with universal screening would be very helpful and much needed.*

[P380, Elementary, 18 years’ experience]

School psychologists emphasized the need for all school staff to be trained in evidence-based practices to improve school safety. They also called for an equitable, systematic, and transparent approach to enhance the effectiveness of school violence prevention and intervention efforts.

#### 3.2.2. School–Family–Community Relationships

The second theme, school–family–community relationships, detailed a need to respond to the strained relationships among educators, families, and students. Specifically, the need to improve the functional relationships among the three groups with consistent and transparent communication to build trust and accountability was mentioned explicitly. One school psychologist described this relationship as a way to enhance school safety:

*Building of community is important for students and families to feel that school is a safe and welcoming environment.*

[P111, Elementary, 25 years’ experience]

Another school psychologist detailed how schools can leverage these relationships to prevent gun violence through conversations about gun safety:

*Parents locking away weapons/limiting access to weapons, and having some real conversations about keeping self and others safe.*

[P194, Elementary, 12 years’ experience]

The subtheme of *school-based mental health* was often referred to as a way to support youth development and their experiences at school and outside of school. One school psychologist listed this subtheme in particular for those with limited access to mental health care (e.g., insurance barriers):

*More mental health resources for our school/community, particularly people who can provide direct services; more consistently followed universal expectations and routines; better accountability and follow-up (of both students and staff); increased communication among staff members.*

[P423, All Grades, 3 years’ experience]

Another school psychologist described the importance of expanding support to families and the invaluable role of school counselors and behavior specialists in schools:

*Continue focus on mental health resources to support students and families. Counselors and behavior specialists in our school buildings are vital.*

[P374, Elementary, 30 years’ experience]

#### 3.2.3. Threat Assessment

The last theme, threat assessment, referred to both formal and informal practices for determining the appropriate procedures for students experiencing a variety of well-known risks for school violence (e.g., aggression, bullying, antisocial behavior, threats). This theme involved a need for all school staff to be trained in threat assessment, the development of threat assessment teams in schools, transparency with the process, and additional support for interventions after a threat has occurred. Many school psychologists called for all staff to be informed on the policies, procedures, resources, and interventions when a school violence threat occurs. One school psychologist underscored the ineffectiveness of exclusionary practices:

*I think there just needs to BE sufficient policies, procedures, resources, and interventions in order to prevent violence in our schools. To my knowledge, there is no actual threat assessment team, and all related issues that come up go directly to law enforcement and the student is suspended or expelled. This is not effective. I think ALL staff needs training, and then there needs to be a very clear “if-then” type chart so all staff knows what to do in different situations.*

[P378, All Grades, 8 years’ experience]

Another school psychologist expressed their need for clarity about the role of mental health assessments in threat assessments:

*We do not have standard procedures when we conduct a mental health assessment as part of a threat assessment. We have a standard threat assessment protocol, but once it gets to the mental health assessment of the individual student by the school psychologist, I feel that we do not have standards to rely on and our training for this is inadequate.*

[P180, Elementary, 13 years’ experience]

Yet another school psychologist explained how in their school, there is crisis prevention institute-based training, but a lack of communication around the procedures in place for a district-level threat assessment team:

*We have most of our staff cpi trained for verbal de-escalation, but not everyone buys into it—we need to convince staff that de-escalation is better than engaging in a power struggle with a 12 year old. We need trauma-informed training, diversity training, and poverty training. We have a district threat assessment team, but there is not much communication from the district about the threat assessments they do and the results—kids just show back up at school upon completion with no communication.*

[P270, Middle School, 22 years’ experience]

Some school psychologists shared responses that indicated they felt generally safe. This subtheme indicated that it was safe in their schools, and there were no solutions that were needed, or they specifically responded with “none”. Similarly, some respondents explicitly stated that they “do not want to answer this question”. The responses below demonstrate the general absence of violence or the type of conflict that the respondents felt would pose a significant safety concern:

*We have regular drills, all outside entrances are locked, we have a liaison with the local police department, parents are supportive. We do not have issues with violence between students, staff or admin.*

[P441, Elementary, 37 years’ experience]

Another psychologist shared:

*We don’t have violent behavior really, other than “typical” fights that occur at the high school level between students”*.

[P196, K–12, 13 years’ experience]

The majority of school psychologists suggested a need for better policies, procedures, resources, and interventions to address and prevent school violence. They specifically mentioned evidence-based training and support with threat assessments, and mental health support, as well as building strong relationships with students and their families.

## 4. Discussion

The findings of this study contribute to the growing body of literature on school safety by centering the perspectives of school psychologists, who are uniquely positioned to assess and address systemic challenges within pre-K–12 educational settings. The three themes from this investigation included (a) aggressive behaviors from students and families, (b) unmet mental and behavioral health needs, and (c) concerns with staffing, underscoring critical vulnerabilities in school safety infrastructure. The three themes representing intervention and prevention strategies included (a) professional development/training, (b) school–family–community relationships, and (c) threat assessment, emphasizing the need for funding and resource allocation to train school staff in evidence-based practices and invest in family partnerships. These insights align with existing research emphasizing the need for structural changes and proactive resource allocation to prevent risks and promote safe and supportive schools. Additionally, this investigation also found emerging subthemes (i.e., families, community factors, staff burnout/role overload and confusion, defiance/disruption, discipline, school-based mental health, and being generally safe) that reflect an array of factors and concerns related to workplace safety from the perspectives of school psychologists. Findings from this study offer valuable insights for future research, school practice, and policies. Specifically, these results have implications for policy development and the allocation of resources to ensure the physical and psychological safety of all students and school staff.

### 4.1. Safety Issues

#### 4.1.1. Aggression from Students and Parents

Our findings suggest that aggression is a major safety concern within schools from the perspective of school psychologists. This concern aligns with research documenting the widespread prevalence of violence in K–12 educational settings and the significant rates reported by school psychologists. McMahon et al. (2024) found that a substantial percentage of U.S. school psychologists, social workers, and counselors (*N* = 2049) experienced verbal and threatened violence from students (63%) and parents (63%) and physical violence from students (50%) and parents (9%) after the COVID-19 restrictions in the 2021–2022 academic year. This study elaborates upon and provides context for a more in-depth assessment of school psychologists’ concerns about aggression from students and parents. These findings also highlight the complex interpersonal dynamics school psychologists must navigate, with aggression stemming not only from students but also from parents, complicating their roles in preventing school violence (McMahon et al., 2023).

The COVID-19 pandemic may have further exacerbated these existing issues, as school psychologist participants reported observing aggressive behaviors, including verbal and physical aggression, following the pandemic. Similarly, Lancaster and Brasfield (2023) found that school counselors reported a rise in aggressive behaviors post-pandemic, reflecting broader concerns among school staff and psychologists about the challenges of managing student behavior as schools transitioned back to in-person interactions after a time of remote or hybrid learning. Most public schools in the U.S. reported that COVID-19 negatively impacted the behavioral development of students; public school students’ aggressive behaviors either increased or remained the same after returning to in-person instruction from COVID-19 (Burr et al., 2024).

#### 4.1.2. Mental and Behavioral Health Concerns

Our findings indicate that school psychologists regard students’ mental and behavioral health issues as significant safety concerns within educational settings. National data show that one in six U.S. youth experiences a mental health disorder each year (National Alliance on Mental Illness, 2024). Given their specialized expertise in mental health and behavioral issues, school psychologists are uniquely positioned to address these challenges (National Association of School Psychologists [NASP], n.d.; Zabek et al., 2023). This expertise likely explains why our results reveal that school psychologists identified mental and behavioral health as critical safety concerns integral to students’ school experiences. However, there remains a significant gap in the delivery of psychological services within schools, given multiple factors, such as meeting student assessment needs, providing teacher support with academic interventions, and managing caseloads across multiple schools (National Association of School Psychologists [NASP], 2024).

Moreover, the existing literature suggests that school psychologists have observed a deterioration in students’ mental and behavioral health following the COVID-19 pandemic (Lancaster & Brasfield, 2023), along with an anticipated increase in demand for mental health services (Schaffer et al., 2021). This shortage, coupled with limited access to external mental health resources, as noted in our results, further complicates the ability of school psychologists to ensure that students receive the support necessary to foster a positive school climate. Additionally, some psychologists pointed out that community factors, such as neighborhood violence, contribute significantly to the mental health challenges faced by students. This perspective underscores the necessity of addressing both school-based and broader socioecological factors to enhance student well-being and maintain a safe educational environment.

Our findings underscore the impact of the COVID-19 pandemic on culture and policy in the macrosystem and on interactions within the microsystem, where students, parents, and school psychologists engage directly with one another (Astor et al., 2023; McMahon et al., 2024). Aggressive behaviors, as well as mental/behavioral health concerns within this immediate environment, can disrupt the educational process and compromise a sense of safety at school, emphasizing the need for targeted interventions that address both individual and environmental factors contributing to aggression in schools.

#### 4.1.3. Staffing Challenges

From the perspectives of school psychologists, our findings further highlight that staffing challenges, including understaffing, inadequate training, and burnout, can negatively impact student mental and behavioral health outcomes and overall school safety. In a qualitative study of school-based practitioners who are NASP members, all participants identified lack of time and heavy caseloads as significant barriers to delivering comprehensive and integrated school psychological services (Castillo et al., 2016). Additionally, they cited limited access to resources, inadequate training, and insufficient professional development as major obstacles (Castillo et al., 2016). From the perspectives of school officials, limited access to licensed mental health professionals and insufficient funding are two primary obstacles that impede schools’ efforts to provide necessary mental health services (Burr et al., 2024; Wang et al., 2020). School counselors have reported similar barriers—that is, high caseloads, a lack of administrative support, and inadequate training—indicating a dearth of the essential resources required to deliver effective services to students (Lancaster & Brasfield, 2023). In contrast to the previously discussed major safety concerns, this issue highlights the preparedness and capacity of school psychologists and other staff to respond effectively to safety challenges. Systemic factors, such as institutional policies and resource allocation, highlighted in Bronfenbrenner’s macrosystems, play a crucial role in elucidating and addressing these safety concerns.

A number of school psychologists felt generally safe in their schools and had no suggestions to improve the safety of their schools. Data do show that overall, schools are becoming safer in terms of face-to-face bullying and sexual violence (Benbenishty et al., 2023; Clayton et al., 2023; Kennedy, 2021), so it is not surprising that some school psychologists reported that their schools are generally safe. It is possible that increased evidence-based violence prevention efforts in schools over decades of school safety research, often involving school psychologists as part of the prevention efforts, are having a meaningful impact on making schools safer (Benbenishty et al., 2023). This study was designed to gather a cross-section of school psychologists in the U.S., and they work in many different contexts. It may be helpful to examine schools where the school staff feel safe, especially in high-risk environments, in order to understand policies and practices that are working well.

### 4.2. Potential Strategies

#### 4.2.1. Training and Support

When prompted to share suggestions to address safety in their schools, school psychologists most often cited the need for more training for themselves and other school staff. School psychologists tended to draw from relevant evidence-based training that emphasizes the importance of social–emotional learning (Chiappetta-Santana et al., 2022; Moral-Bofill et al., 2015), trauma-informed approaches (Ormiston et al., 2020), and culturally sensitive strategies (Grant et al., 2022; Parker et al., 2021). Such suggestions are in keeping with currently accepted best practices to address school safety and foster a positive school climate in various communities (VanLone et al., 2019; Voight & Nation, 2016). School psychologists are often tasked with implementing schoolwide climate and safety programming in practice (Bradshaw et al., 2021), and they draw on perspectives that emphasize the inclusion of diverse backgrounds and family-centered approaches (Grant et al., 2022; Sheridan & Garbacz, 2022).

School psychologists mentioned the need for more training for themselves and other school staff on addressing safety and climate. School psychologists may feel more knowledgeable about behavioral and emotional concerns than other school staff who are primarily tasked with academically supporting students. Research on teacher perspectives suggests an ecological approach to training is needed to prevent school violence toward educators and students (McMahon et al., 2020). A comprehensive and multi-tiered school support system for school safety would require higher competencies from all school staff to be able to play a productive role in ensuring school safety (Cowan et al., 2013; VanLone et al., 2019). It is evident that all school staff, students, and families are responsible for fostering and maintaining a safe school community.

#### 4.2.2. Evidence-Based Practices

Threat assessment was a popular suggestion among our participants. As of the 2022–23 school year, 82% of U.S. public schools report having a behavioral threat assessment team or similar group (Burr et al., 2024). A comprehensive threat assessment team would include a school psychologist who can provide support by performing many different roles, including determining necessary assessments, collecting data from relevant stakeholders, keeping and reviewing records, and advising on additional supports for students (Kelly, 2018). There are mixed results in terms of the effectiveness of threat assessment teams in schools, with particular concerns around perpetuating disparities in discipline based on race, gender, and abilities (Crepeau-Hobson & Leech, 2022), though D. Cornell and colleagues (2018) have shown that culturally aware threat assessment can enable more non-disparate outcomes (Maeng et al., 2024).

School-based mental health was also identified as a practice and policy for school safety by school psychologists, particularly around the lack of resources in terms of prevention. The wide-ranging positive benefits of school-based mental health to student functioning have long been identified (Owens & Murphy, 2004), with more effective programs involving targeted and selective prevention (Sanchez et al., 2018), which naturally would require more investment in and involvement from school psychologists. Safer schools are built by forming safe relationships (Eppler-Wolff & Martin, 2021), which require the recognition of emotional development and mental health among both school psychologists and other school staff that students form bonds with. It is most beneficial to have school staff, such as teachers (Dinnen et al., 2024), administrators (Frey et al., 2022), and counselors (Christian & Brown, 2018), with mental health knowledge and evidence-based practices to implement school-based mental health programming (National Association of School Psychologists [NASP], 2021).

Our findings highlight the importance of training needs and evidence-based practices within the microsystem, where school psychologists and staff must adhere to designated safety plans and pursue ongoing professional development. School psychologists also emphasize potential solutions within the exosystem, where support from educational and organizational structures (e.g., district, county, and federal entities) plays a crucial role in providing adequate resources and prioritizing school safety (Astor et al., 2023; Espelage, 2014). These results reinforce the recommendation for coordinated efforts across social ecologies to enhance school safety and foster a supportive educational environment.

#### 4.2.3. Effective Discipline

Many school psychologists emphasized a need for effective discipline in terms of both holding students accountable for repeated behaviors and transgressions, as well as holding other school staff, school administrators, and families accountable, to better support students. McMahon et al. (2023) found that teacher safety concerns related to parental aggression were often related to school discipline practices. Some school psychologists advocated for restorative justice and trauma-informed approaches, while others advocated for hardening consequences, such as more detentions/suspensions and removals of students who exhibit repeated behaviors that are harmful or very disruptive to others. Zero-tolerance policies and practices have been found to be disproportionately ineffective and iatrogenic for students of color in U.S. schools (Espelage et al., 2023; Fadus et al., 2021). Restorative justice, although widely studied in the criminal justice field, has been only recently rigorously studied in educational settings (Darling-Hammond, 2023; Gregory et al., 2022). Additional research is needed to inform the implementation of effective discipline.

Whether school psychologists were advocating for a hardening or more nuanced restorative or trauma-informed approach, they shared that a comprehensive, consistent approach to discipline was needed to better mitigate risk and restore order and safety at school through improving trust and connection in the mesosystem (Espelage, 2014). Their perspectives also highlight the importance of incorporating diverse backgrounds and lived experiences into disciplinary practices, reflecting a broader belief across social disciplines.

### 4.3. Implications for Research, Practice, and Policy

Our findings provide implications that can inform school psychologists’ professional practices and school psychology research to cultivate a safer school environment. In managing aggressive behaviors, our results emphasize the necessity of training and evidence-based practices for early detection of and intervention in school violence. Engaging parents in safety training and awareness initiatives is also crucial, as school psychologists identified aggressive behaviors from students and families as major concerns. A comprehensive and consistent approach to disciplinary practices is needed to ensure clear expectations while reducing disciplinary disagreements, which often serve as a primary source of school–family conflicts (McMahon et al., 2014). Moreover, because school psychologists may not always be in close contact with students, evidence-based strategies that involve other school staff are vital for effectively managing aggressive behaviors. Given the paucity of relevant literature, we recommend that future research explore aggressive behaviors from parents against school staff and examine the school–family dynamics contributing to safety issues.

To support students’ mental and behavioral health, U.S. government funding and resources must be directed toward expanding school-based mental health programming (Salerno, 2016). Additionally, a shift in educational goals is critical, one that emphasizes the importance of social–emotional development alongside academic goals. To achieve this, a reduction in high-stakes testing may alleviate stress for students, staff, and families, allowing schools to focus more on holistic student well-being. Because school psychologists are one of the primary agents in addressing mental and behavioral health issues within schools, active communication and collaboration with other school staff and administrators are essential for creating impactful and lasting school climate improvements. This partnership is particularly beneficial for developing strategies that inform practice and enhance school-based mental health policies. Further research would benefit from conversations with school psychologists to identify challenges and solutions for the development, implementation, and evaluation of school safety initiatives to address the research–practice gap.

Addressing understaffing issues in U.S. schools is also critical, as it directly impacts school psychologists’ ability to meet students’ needs effectively and has negative consequences for school safety. Increased funding and the reallocation of school-level resources are essential not only for hiring additional staff to alleviate overburdened caseloads but also for enhancing training in areas such as SEL, trauma-informed care, threat assessment, and restorative justice for all relevant school staff. By providing enhanced support in these areas, schools can foster a safer and more supportive environment for students, families, and staff. Moreover, research into how school psychologists’ experiences of burnout, job satisfaction, and the availability of resources impact school safety warrants further investigation.

### 4.4. Strengths and Limitations

One of the key strengths of the present study is the use of a national sample of school psychologists. By recruiting participants from diverse regions, school levels, and urbanicity, the study captures a range of experiences and perspectives related to school safety concerns across the U.S. Additionally, the qualitative methodology employed in this study allowed for an in-depth exploration of the nuanced challenges school psychologists face regarding safety in schools. Open-ended survey questions provided participants the opportunity to express concerns and suggest solutions in their own words, offering richer insights than would be possible through purely quantitative data. By using a socioecological framework to organize the findings, the study captures the interconnectedness of safety issues and solutions across multiple levels of influence, from the individual to the broader social and cultural context. Lastly, the rigorous coding process, involving both inductive and deductive approaches and a high inter-coder reliability (>80%), contributes to the credibility and trustworthiness of the findings. The use of multiple coders and a consensus-based approach to resolving discrepancies further enhanced the reliability of the thematic analysis.

Despite its strengths, this study has several limitations. One limitation is that these findings are specific to the U.S. sociocultural context and may not be generalized to other societies. Another limitation is that even though the survey was not intended to focus on school psychologists who experienced violence, those who responded to the survey may have been more likely to have experienced violence than those who did not complete the survey. Further, those who responded to the open-ended questions may also have had different viewpoints from school psychologists more generally. Although our sample was sizable, we were unable to explore the school safety concerns of school psychologists in different career stages and with different training backgrounds, as well as those who have diverse backgrounds and identities. Future studies are needed to fully understand the varied perspectives of school-based mental health professionals.

## 5. Conclusions

School safety remains a significant public health concern in the U.S., and school psychologists are critical in developing warm, supportive, and safe learning environments. The present study explored school psychologists’ perspectives on (a) safety issues facing their schools and (b) violence prevention policies and procedures deemed to be most effective. The safety concerns shared by school psychologists suggest a complex, interconnected system of challenges ranging from individual student and parent issues to school-wide staffing and preparedness issues. As a result, there is a need to increase evidence-based safety training in schools and to involve students and families. A person-centered socioecological model (Espelage, 2014) as a framework can be helpful in developing actionable steps toward change. As efforts in improving school safety are at the forefront of youth violence and injury prevention, studies should continue to amplify the voices and interventions of school psychologists.

## Figures and Tables

**Table 1:** Demographic characteristics of participants.

**Gender**	**Number**
Female	462
Male	74
Multiple	1
Non-Binary	1
**Race**	**Number**
White	419
Multiracial	51
Hispanic	30
African American	21
Asian	9
Other Race	4
Unknown	4
**Urbanicity**	**Number**
Suburban	264
Urban	180
Rural	94
**Years’ Experience**	**Number (years)**
Mean	13.57
Median	11.00
Range	2–41
Standard Deviation	9.20
**School Level**	**Number**
All Grades (Pre-K–12)	73
Elementary School (Pre-K–6)	231
Middle School (5–9)	89
High School (9–12)	107
Unknown	38
**Region**	**Number**
West	127
Midwest	138
South	125
Northeast	115
Alaska	1
Hawaii	1
Puerto Rico	1
Unknown	30

## Data Availability

The data are not currently available for public access due to the size of the datasets, which are currently undergoing ongoing data cleaning, organizing, scale refinement, and scale validation. The study materials are available upon request.
